# Germline thymidylate synthase deficiency impacts nucleotide metabolism and causes dyskeratosis congenita

**DOI:** 10.1016/j.ajhg.2024.05.022

**Published:** 2024-06-11

**Authors:** Hemanth Tummala, Amanda Walne, Roberto Buccafusca, Jenna Alnajar, Anita Szabo, Peter Robinson, Allyn McConkie-Rosell, Meredith Wilson, Suzanne Crowley, Veronica Kinsler, Anna-Maria Ewins, Pradeepa M. Madapura, Manthan Patel, Nikolas Pontikos, Veryan Codd, Tom Vulliamy, Inderjeet Dokal

## Main text

(The American Journal of Human Genetics *109*, 1472–1483; August 4, 2022)

The image in panel B of Figure 1 has been amended at the request of the parents of family 1.

